# Lambeau grand dorsal dans la reconstruction d’une tumeur géante de la paroi abdominale: à propos d’un cas rare

**DOI:** 10.11604/pamj.2017.27.181.11028

**Published:** 2017-07-06

**Authors:** Karim Bourra, Samir El Mazouz

**Affiliations:** 1 Service de Chirurgie Plastique, Hôpital Al Farabi Oujda, Maroc; 2 Service de Chirurgie Plastique, Hôpital Avicenne, Rabat, Maroc

**Keywords:** Musculo-skin flap, latissimus dorsi flap, free flap, microsurgery, termino lateral anastomosis, Musculo-skin flap, latissimus dorsi flap, free flap, microsurgery, termino lateral anastomosis

## Abstract

Nous nous permettons de vous présenter le cas d’un jeune patient, âgé de 16 ans, qui présente une grosse tumeur de la paroi abdominale, multinodulaire de nature mésenchymateuse occupant la moitié abdominale gauche et mesurant dans les 25 cm d’axe vertical / 20 cm d’axe transversal, mobile par rapport aux plans profonds, et augmentant progressivement de volume depuis l’enfance et négligée. Nous avons dans le but du traitement chirurgical, après petite exérèse biopsique qui a révélé la nature «desmoide » de la tumeur, réalisé une exérèse chirurgicale total de la tumeur avec reconstruction immédiate par un lambeau musulo-cutané de grand dorsal libre, branché sur les gros vaisseaux du pli de l’aine (artère iliaque et veine iliaque externes gauches), branchés en anastomose termino-latérale. La survie du lambeau s’est faite correctement et la reconstruction a ainsi été réussie.

## Abstract (en)

We report the case of a 16-year old patient presenting with giant, multinodular, mesenchymal tumor of the abdominal wall occupying the left abdominal region and measuring 25 cm on the vertical axis, 20 cm on the transverse axis, mobile when compared with the deep structures and gradually increasing in volume over childhood and neglected. After small biopsy, which showed desmoid tumor, the patient underwent complete surgical resection of the tumor with immediate reconstruction by free muscolo skin flap of the latissimus dorsi attached to the large blood vessels of the inguinal fold (left iliac artery and left external iliac vein), connected by termino lateral anastomosis. Flap survival was correctly performed and reconstruction was successful.

## Introduction

**Figure 1:**
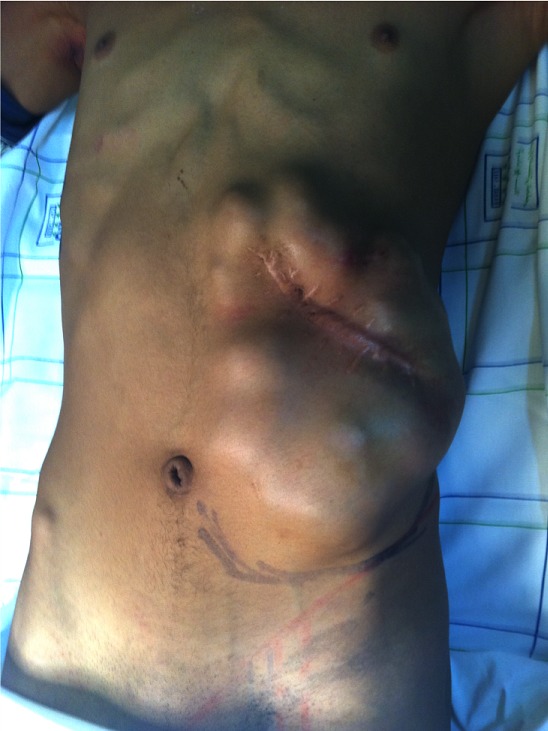
Vu de la tumeur géante occupant une large partie de l’abdomen

**Figure 2:**
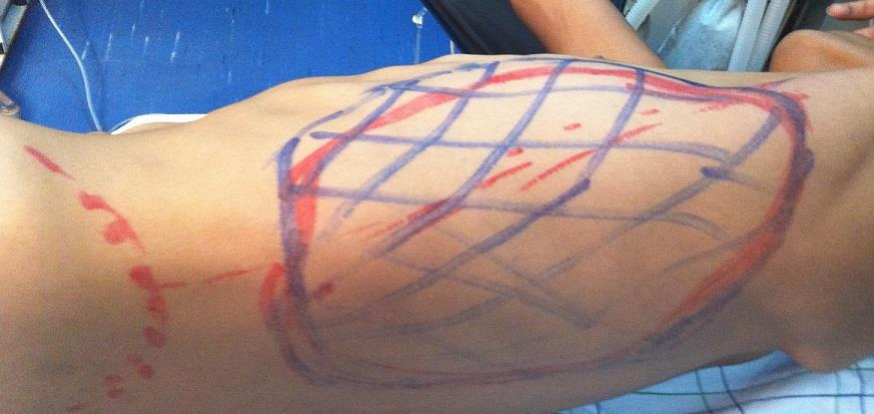
Dessin de la palette cutanée du lambeau grand dorsal utile identique à celle mesurée sur la surface de la tumeur

**Figure 3:**
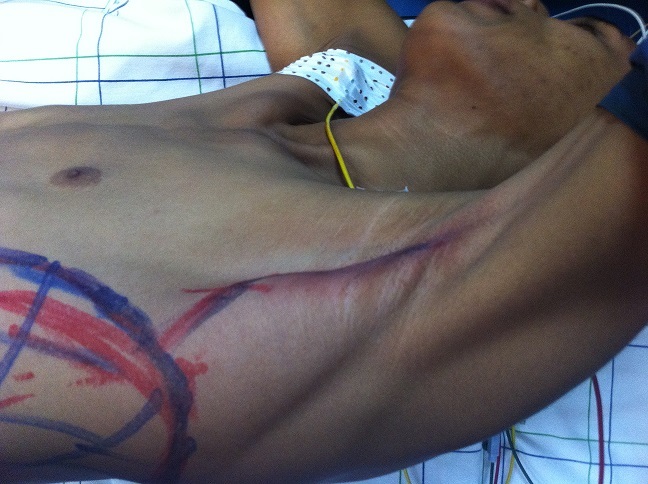
Vu du prolongement axillaire du pédicule thoraco-dorsal vascularisant le lambeau du grand dorsal

Nous présentons le cas d’un jeune patient, âgé de 16 ans, qui présente une grosse tumeur de la paroi abdominale (Figure 1), dont l’exérèse large a nécessité le recours au lambeau musculo-cutané de grand dorsal, prélevé libre de ses vaisseaux et branché aux gros vaisseaux du pli de l’aine pour la couverture définitive et correcte de cette perte de substance abdominale (tout en un temps chirurgical) [1]. Avec un premier temps chirurgical, d’exérèse tumoral (Figure 2, Figure 3) puis un deuxième temps chirurgical de lambeau libre, prélevé en changeant de position puis un troisième temps chirurgical et dernier temps, de microchirurgie, pour brancher le pédicule thoraco-dorsal du muscule (artère et veine) aux vaisseaux inguinaux (artère et veine iliaques externes) [2].

## Patient et observation

Notre cas rare de tumeur abdominale géante, multinodulaire de nature fibromateuse développée aux dépends des muscles de la paroi abdominale, apparaissant chez un garçon âgé de 16 ans, touchant tout le cadrant abdominal gauche et intéressant la moitié de l’hypochondre gauche, tout le flanc gauche ainsi que le pli de laine gauche. La tumeur a évoluée à bas bruit depuis des années en augmentant progressivement de volume. Le bilan biologique ne montre aucune anomalie particulière. Le bilan radiologique a consisté en une échographie abdomino-pelvienne et un scanner thoraco-abdominal avec et sans injection de produits de contraste. L’échographie a montré une masse tumorale hétérogène hyperéchogènes avec des zones hypoéchogènes. Les masses tumorales sont encapsulées. La tumeur est mobile par rapport au plan profond et libre de toute adhérence profonde. Le scanner abdomino-pelvien réalisé a montré la masse tumorale multinodulaire constituées de nodules encapsulés développés aux dépens des tissus musculaires de paroi abdominale. Une petite exérèse biopsique a permis de confirmer le diagnostic de « tumeur desmoide » [2].

### Diagnostic différentiel

Cette forme immature pose un diagnostic différentiel crucial, avec un rhabdomyosarcome embryonnaire [3–5]. L'analyse morphologique très scrupuleuse de la prolifération notant l'absence d'anomalies cytologiques, la rareté des mitoses, la répartition homogène des éléments cellulaires doivent être pris en compte, ainsi que l’immunophénotype. Le diagnostic différentiel se pose aussi avec un fibrosarcome infantile, certains critères doivent être particulièrement pris en compte, tel que le caractère infiltrant, l'absence d'anomalie cytologique, la présence d'une chromatine fine claire, la rareté des mitoses (inférieure à 3 mitoses pour 10 grands champs), sont des éléments en faveur de la fibromatose.

### Caractères évolutifs

**Figure 4:**
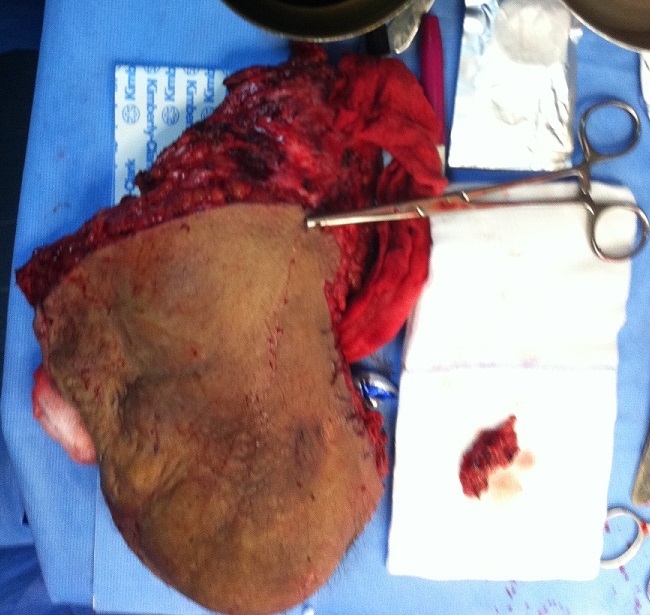
Vu per opératoire de la pièce de résection

**Figure 5:**
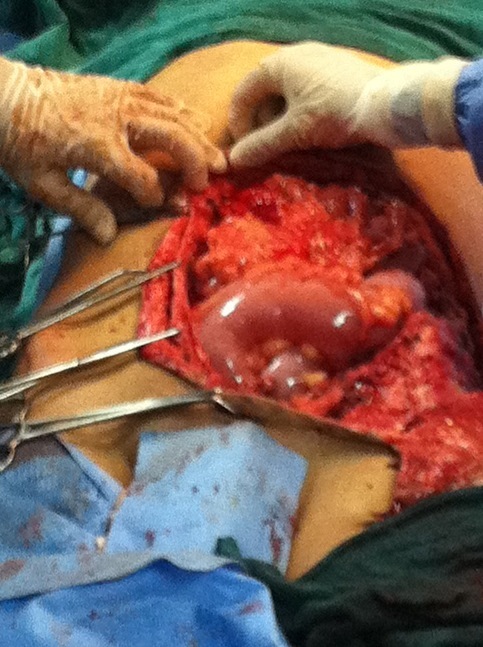
La perte de substance ainsi obtenue après l’exérèse tumorale

Bien que ces tumeurs ne donnent pas de métastase, elles ont une grande tendance à récidiver si l'exérèse initiale n'a pas été satisfaisante. Le traitement de choix est une exérèse satisfaisante la plus large possible (Figure 4, Figure 5). Bien que l'étiopathogénie ne soit pas claire [6, 7], un traumatisme a pu être évoqué dans certains cas.

## Discussion

**Figure 6:**
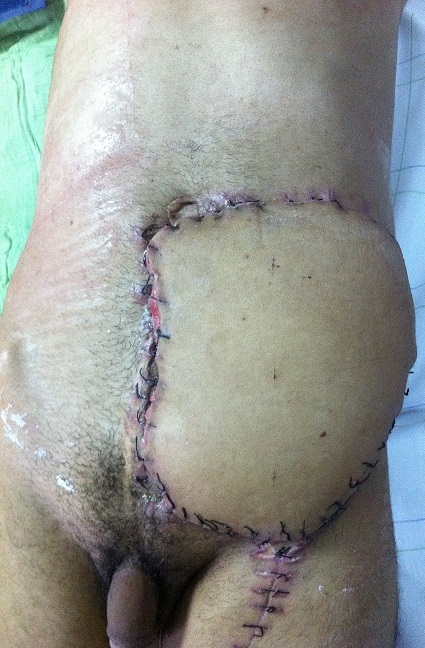
Vu en post-opératoire du lambeau mis en place et vivant à J3 post-opératoire

**Figure 7:**
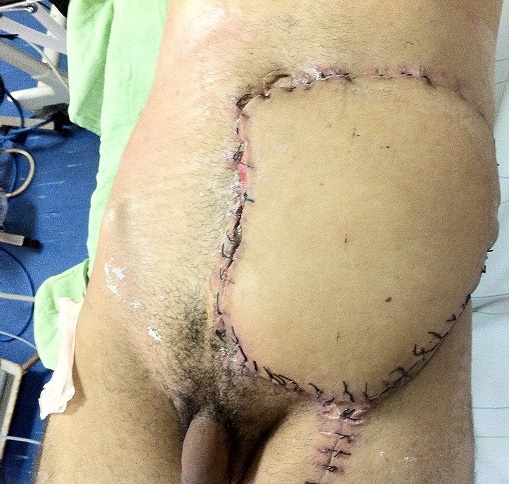
Vu de la paroi abdominale reconstruite par notre lambeau sans souffrance cutanée ni nécrose (patient mis sous ATC pendant 7 jours)

Nous discutons la reconstruction de la perte de substance de la paroi abdominale par l’utilisation de greffe de peau mince après bourgeonnement de la perte de substance de la paroi abdominale 4 semaines après l’exérèse de la tumeur abdominale initiale et cicatrisation dirigée. Par opposition à la mise en place d’un lambeau libre du muscle grand dorsal homolatéral prélevé libre avec son pédicule (Figure 6, Figure 7) et branché par anastomose termino-latérale sur les vaisseaux inguinaux homolatéraux [8, 9]. Après étude des avantages et inconvénients des deux méthodes, nous concluons que la technique du lambeau grand dorsal libre branché [10], présente un avantage certain à celle de greffe de peau mince pour un meilleur résultat esthétique et fonctionnel pour le patient (Tableau 1).

**Tableau 1:** Comparatif entre GPM et LGD avantages/inconvénients

	Greffe de peau mince	Lambeau grand dorsal
**Avantages**	- Facile à prélever - Cicatrisation rapide - Guérison rapide	- Fiable - Facile à prélever - Vaisseaux fiables - Microchirurgie facile - Résultat constant
**Inconvénients**	- Cicatrice indélébile - Cicatrice en carreaux si greffe en maille de filet	- Marche avec béquilles si nécessaire difficile - Surface du lambeau limité - Possibilité d’échec d’anastomose

Abréviations: ATC = anticoagulants

## Conclusion

Notre étude comparative a permis de faire le choix de la fiabilité et de l’esthétique en utilisant le lambeau du grand dorsal libre branché grâce à son pédicule aux vaisseaux iliaques externes. Notre lambeau mis en place a permis la couverture totale de la perte de substance abdominale obtenue après exérèse complète de la volumineuse tumeur abdominale. Résultat post-opératoire satisfaisant après reconstruction réussie de la paroi abdominale en trois temps opératoires (Tout en un temps) mais dont la durée assez longue constitue l’inconvénient majeur. Le patient fut suivi à 3 mois, 6 mois et un an avec un bilan post-opératoire rapproché et un bilan d’extension tumoral pour détecter à temps une éventuelle récidive locale.

## Conflits d’intérêts

Les auteurs ne déclarent aucun conflit d'intérêt.
